# Mast Cells and Histamine: Do They Influence Placental Vascular Network and Development in Preeclampsia?

**DOI:** 10.1155/2012/307189

**Published:** 2012-06-19

**Authors:** Grzegorz Szewczyk, Michał Pyzlak, Jakub Klimkiewicz, Wacław Śmiertka, Magdalena Miedzińska-Maciejewska, Dariusz Szukiewicz

**Affiliations:** ^1^Chair and Department of General and Experimental Pathology, Medical University of Warsaw, Ul. Krakowskie Przedmiescie 26/28, 00-927 Warsaw, Poland; ^2^Department of Gynecological Oncology, National Institute of Oncology, Ul. Wawelska 15, 02-000 Warsaw, Poland; ^3^Department of Anesthesiology and Intensive Care, Military Institute of Health, Ul. Szaserów 128, 04-141 Warsaw, Poland

## Abstract

The physiological course of pregnancy is closely related to adequate development of the placenta. Shallow invasion of trophoblast as well as decreased development of the placental vascular network are both common features of preeclampsia. To better understand the proangiogenic features of mast cells, in this study we aim to identify the potential relationship between the distribution of mast cells within the placenta and vascular network development. *Material and Methods*. Placentas from preeclampsia-complicated pregnancies (*n* = 11) and from physiological pregnancies (*n* = 11) were acquired after cesarean section. The concentration of histamine was measured, and immunohistochemical staining for mast cell tryptase was performed. Morphometric analysis was then performed. *Results*. We noticed significant differences between the examined groups. Notably, in the preeclampsia group compared to the control group, we observed a higher mean histamine concentration, higher mast cell density (MCD), lower mean mast cell (MMCA) and lower vascular/extravascular (V/EVT) index. In physiological pregnancies, a positive correlation was observed between the histamine concentration and V/VEVT index as well as MCD and the V/VEVT index. In contrast, a negative correlation was observed between MMCA and the V/EVT index in physiological pregnancies. *Conclusions*. Based on the data from our study, we suggest that a differential distribution of mast cells and corresponding changes in the concentration of histamine are involved in the defective placental vascularization seen in preeclamptic placentas.

## 1. Introduction

Angiogenesis is a crucial process for the growth and development of new tissues. We can observe angiogenesis in neoplasms, during tissue repair after injury and in the placenta. Proper placental angiogenesis is necessary for the normal course of pregnancy and labor [1]. The pathogenesis of preeclampsia is still unclear, but it is known that shallow spiral artery invasion may contribute to preeclampsia development. Shallow spiral artery invasion results in poor placental perfusion and may lead to hypoxic stress in the fetus. Immaturity of extravillous trophoblastic cells has been identified as a cause of diminished spiral artery invasion [2]. The placental vascular network is defectively developed as well. In some preeclampsia-complicated pregnancies, the placenta and associated placental vascular network are diminished. Mast cells are found in the placenta in every stage of placenta development. Their potential role, apart from immunological properties, can be associated with proangiogenic activity. Mast-cell-derived mediators of known angiogenetic potential include vascular endothelial growth factor (VEGF), transforming growth factor beta (TGF-*β*), histamine, tumor necrosis factor alpha (TNF-*α*), interleukin-8, and basic fibroblast growth factor [3]. The activation and degranulation of mast cells in the place of angiogenesis stimulate vessel sprouting and sustain mast cell attraction and activation [4]. Data from the literature and our own experience suggest that mast cells may be involved in the pathogenesis of preeclampsia-complicated pregnancies [5, 6]. In this study, we examined the relationship between mast cells (number and morphological features), histamine concentration, and microvascular density in placentas obtained after delivery from normal and preeclampsia-complicated pregnancies. 

## 2. Material and Methods

The characteristics of the patients are detailed in Table 1. Placental samples were obtained in a standardized manner after the dissection of fetal membranes. Three samples were excised from the maternal side of the placenta and two were excised from the fetal side. Macroscopically changed areas, large vessels, and fibrous tissues were avoided. Samples were taken immediately after cesarean sections in each group: preeclamptic women (PE, *n* = 11) and healthy women (control group, *n* = 11). In the PE group, cesarean sections were performed due to severe preeclampsia. In the control group, cesareans were performed due to severe myopia and breech presentation of the fetus. None of the patients included in the study had contractile activity [7]. The study was reviewed and accepted by the local ethic committee. 

## 3. Immunocytochemical Stainings

The tissue fragments were fixed in formaldehyde solution, dehydrogenized with 96% alcohol, acetone, and xylene and then paraffinized. Next, they were cut in microscopic slides and deparaffinized, and the intrinsic peroxidase activity was blocked with hydrogenium superoxide. The samples were then washed with PBS and incubated with normal human serum for 20 minutes. Excess antibody was removed, and the slides were incubated with mouse anti-tryptase antibody (Novocastra, 1 : 3000), followed by secondary anti-mouse biotinylated antibody and Novostain Super ABC Reagent (Novocastra). Both incubations lasted for 30 minutes. The slides were washed with PBS and exposed to 3,3′-diaminobenzidine (Immunotech) for 3 minutes as an electron donor and hydrogen peroxide as a substrate, resulting in a brown reaction product. The cells were then counterstained with Mayer's hematoxylin (Sigma) for 1 minute. Finally, the slides were mounted with DPX (Sigma). As a negative control, the slides were incubated with PBS instead of the primary antibody.

## 4. Histamine Concentration Assay

A fluorimetric method was applied as previously described [8]. The determination of histamine was based on a precolumn derivatization with o-phthaldialdehyde using reversed-phase high-performance liquid chromatography in perchloric acid extracts. A fluorescence detection system was used, with the excitation set at 360 nm and the emission read at 455 nm. The intra- and inter-assay coefficients of variation were 8.5% and 10.0%. 

## 5. Morphometric Analysis

**Figure 1:**
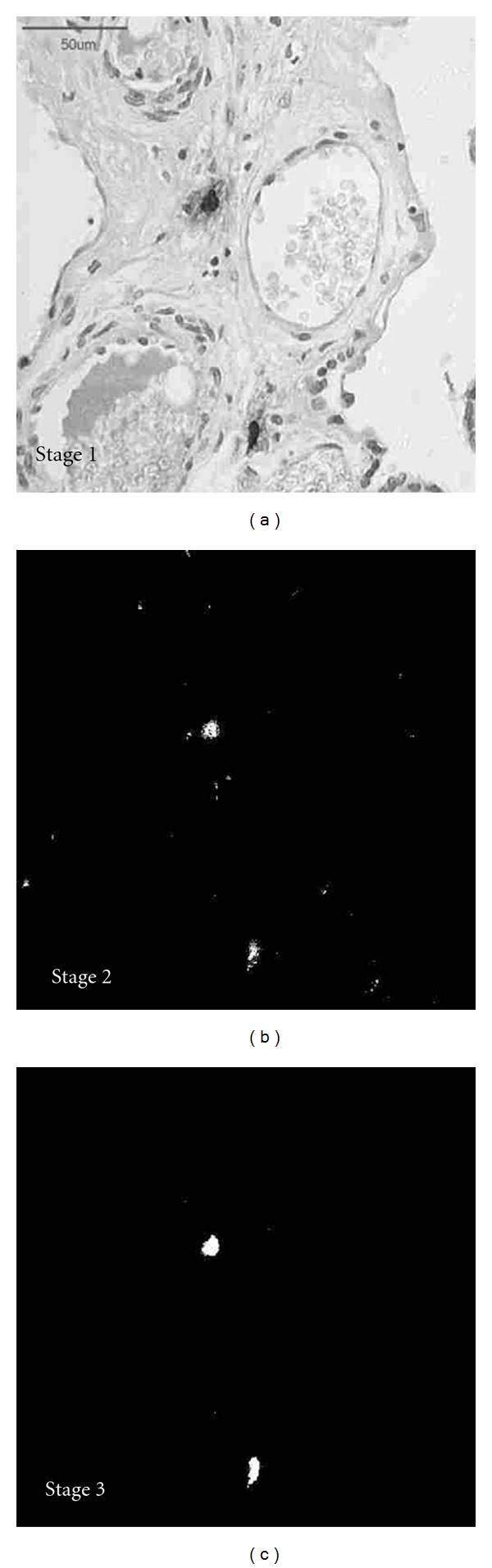
Process of mast cells identification with morphometric software. Stage 1: initial picture obtained from microscope and saved in HSI colour system. Stage 2: binary image after detection of mast cells in distinct hue values. Stage 3: final reduction of noises and smoothing of detected fields.

Morphometric analysis was carried out with the computer image analysis system Leica Quantimet 500C+ (Leica Cambridge Ltd. Cambridge, UK). The system consisted of an IBM Pentium computer operating at 120 MHz equipped with an ARK Logic 2000MT graphic card and graphic processor. The computer was connected to a CCD video camera JVC TK-1280E and Leica DMLB light microscope. Sections of placentas were imaged using a 20 : 1 objective and a 10 : 1,20 ocular. The optical image was focused by a video camera, and an analogue video signal was generated. An analogue to digital converter (ADC) produced a digitized video with distinct color level values in HSI system. The images were processed, and mast cells and placental vessels were clearly identified [9] (Figure 1).

Two independent researchers were responsible for image acquisition and analysis. All measurements were recorded in a blinded fashion. Neither researcher had previous knowledge of the clinical data. For each case, 50 random visual fields were analyzed. After system calibration, the area of a single analyzed image (visual field) was defined as approximately 0,14 mm^2^. The following parameters were analyzed: mast cell density (MCD), defined as number of mast cells per mm^2^ of placental tissue; mean mast cell area (MMCA), the mean area of mast cells cross-sections; shape of mast cells, defined as the ratio of long to short axis of a cell (with perfectly round cells defined as having 1.00 index); vascular/extravascular tissue index (V/EVT index), the ratio of vessel cross-section area to remaining placental tissue. Technical error caused by uniaxial sections of vessels was eliminated by accepting the lowest value of Ferret's diameter as the diameter for a single lumen. Vessels between 10 and 70 *μ*m in diameter were included for analysis.

## 6. Statistical Analysis

Statistical analysis was performed with Statistica 8.0 (StatSoft, Poland). Groups were compared with Student's *t*-test. In each group analysis, correlation was measured between the histamine concentration, V/EVT index, and morphometric parameters of the mast cells. Differences were deemed statistically significant if *P* < 0, 05. 

## 7. Results

Specific differences were observed in several examined parameters between the PE and control groups. The mean histamine concentration (ng of histamine per 1 g of tissue) was significantly higher in the PE group compared to the control group (245,6 ± SD 19,8 versus 175,1 ± SD 15,1; *P* = 0, 002). MCD (in cells/mm^2^) was also significantly higher in the PE group compared to the control group (7,67 ± SD 3,56 versus 2,89 ± SD 1,34; *P* = 0, 004). In contrast, the MMCA was significantly lower in the PE group in comparison to the control group (62,25 *μ*m^2^ ± SD 18,91 versus 101,98 *μ*m^2^  ± SD 57,91; *P* = 0, 0428). We also observed some differences in cell shape. Mast cells in the control group were longer than mast cells in the PE group (shape index 1,88 ± SD 0,8 versus 1,52 ± SD 0,39; *P* = 0, 051; refer to Table 2).

Morphometric assessment of placental circulature was performed and revealed a decrease in the V/EVT index in the PE group compared to the control group (0,15 ± SD 0,04 versus 0,23 ± SD 0,074; *P* = 0, 005; refer to Table 2). 

The analysis revealed a positive correlation between the histamine concentration and the V/VEVT index as well as between MCD and the V/VEVT index. A negative correlation existed between the MMCA and V/EVT index in the control group, while the PE group showed no significant correlation between these parameters. Specific values of correlation for these parameters are provided in Table 3.

## 8. Discussion

Angiogenesis is the process of vessel growth from preexisting vessels, a process that requires stimulation by proangiogenic factors. Important stimulants of placental angiogenesis include VEGFs and placental growth factor, which act through the VEGF receptor family. VEGF production is stimulated by histamine acting through the H_2_ receptor [10]. Mast cells are pointed to as a potential source of potent proangiogenic factors during angiogenesis, including histamine, VEGF, bFGF, TGF-beta, TNF-alpha, and IL-8. Additionally, mast cells are a source of extracellular matrix-degrading proteinases [4]. 

In vitro models of angiogenesis observed in hypoxic conditions provide us with information on increased angiogenesis, which occurs mainly through increases in VEGF synthesis [11]. Histamine proangiogenetic action is provided through H_1_- and H_2_-receptor-mediated VEGF synthesis. Mast cell degranulation leads to a local increase in histamine concentration and therefore an increase in VEGF synthesis. Mast cells, however, synthesize and secrete VEGF apart from histamine. The final effect is vigorous formation of new vessels in place of mast cell degranulation [12, 13]. 

Decreases in mast cell density in connection with decreased histamine concentration correlated with lower V/EVT index values; nevertheless, this correlation was observed only in the control group. Decreased mast cell area may indicate changes in mast cell activation, perhaps as an effect of degranulation. Hypoxia, which is dominant during placenta formation, is a potent stimulator for mast cell activation and new vessel formation. The most important pathway through which hypoxia stimulates angiogenesis is the activation of hypoxia inducible factor-1*α* (HIF-1*α*) transcription and further synthesis of VEGF. It is also observed that the synthesis of histamine within mast cells and their degranulation is increased after stimulation with HIF-1*α* that is achieved through histidine decarboxylase (HDC, EC:4.1.1.22) [14].

Preeclampsia is a specific state of pregnancy associated with hypertension and proteinuria. Shallow trophoblast invasion of maternal spiral arteries results in an increase in systemic blood pressure. The leading hypothesis for preeclampsia pathogenesis suggests it may arise in order to maintain placental perfusion pressure at a satisfactory level [15]. The vascular bed of the placenta is diminished as a whole, with reduced branching and malformations observed; blood vessels are characterized by decreased number, lumen diameter, and total lumen area [16]. Data from our study support this previous finding, as the V/EVT index was decreased in the PE group compared to the control group. The reduced proportion of vascular area may reflect diminished placental angiogenesis in the first trimester of pregnancy. The decreased vascular network development is a result of a multifactorial pathogenetic course as well as inherited conditions.

The differences in mast cell organization observed between the PE and control groups suggest that mast cells take part in the process of vessel development. Because mast cells are observed to gather close to blood vessels just before the process of angiogenesis begins (this is particularly characteristic for neoplasm growth [17]), we expect an expanded vascular network in preeclamptic placentas. In our study, we observed an increase in mast cell density and an increase in histamine concentration but a low V/EVT ratio. We conclude that in PE, susceptibility to histamine and/or other mast cell proangiogenic compounds may be decreased. In PE placentas, the mast cells had a different shape and smaller area in comparison to the control group. The data suggest that we observed mast cells after an intensive degranulation, as we also found an increased concentration of histamine [18]. Increased mast cell density and histamine concentration can be a compensation effect for incorrect vascular network development. On the other hand, we cannot exclude impairments in histamine receptor configuration. Functional predominance of intracellular histamine receptor (H_IC_) over H_1_ and H_2_ receptors may be a causative factor in the observed decreased angiogenesis [19]. 

The reason for the decreased V/EVT index in preeclamptic placentas may be associated not only with decreased angiogenesis but also with fibroblast proliferation and fibrosis in the extravascular area. In the examined material, the V/EVT index was assessed in placentas obtained during the third trimester. A remodeling of extravascular tissue during the pregnancy should also be taken into consideration. Mast cells are sources of matrix-degrading enzymes including collagenases and gelatinases [4]. Prolonged stimulation of mast cells with hypoxia leads to an increase in collagenolytic activity and an accumulation of low molecular collagen fragments, thus providing a stimulatory factor to fibroblasts and smooth muscle cell proliferation [20]. A dominance of activated fibroblasts may lead to a decrease in the V/EVT index.

We conclude that mast cells are strongly involved in the pathogenesis of preeclampsia, as their concentration and activity are changed in preeclamptic placentas in comparison to physiological placentas. Low vascularization in preeclamptic placentas despite higher histamine concentration and accumulation of mast cells suggests that mast cells fail in their proangiogenic potential, concurrently increasing extravascular activity.

## Figures and Tables

**Table 1:** The characteristics of patients included into the study.

	PE group *n* = 11		Control group *n* = 11	
	Median	Range	Median	Range
Mother's age	30	27–42	30	23–37
Weeks of gestation	37,5	35–40	39	37–40
Birth weight (g)	2485	1650–3650	3400	2630–3810
1st minute Apgar's score	9,5	3–10	10	9-10

**Table 2:** Morphometric parameters analyzed in the study compared with Student's *t*-test. V/EVT index: vascular/extravascular tissue index, MCD: mast cell density, and MMCA: mean mast cell area. The statistically significant results are in bold.

	PE group		Control group		
	mean	SD	mean	SD	*P*
V/EVT index	**0,15**	**±0,04**	**0,23**	**±0,074**	**0,005**
MCD (n/mm^2^)	**7,67**	**±3,56**	**2,89**	**±1,34**	**0,0004**
MMCA (*μ*m^2^)	**62,25**	**±18,91**	**101,98**	**±57,91**	**0,0428**
Shape index	1,52	±0,39	1,88	±0,8	0,051

**Table 3:** The indexes of correlation between histamine concentration and morphometric parameters of mast cells and V/EVT index in each group. V/EVT index: vascular/extravascular tissue index, MCD: mast cell density, MMCA: mean mast cell area. The statistically significant results are in bold.

	PE group *n* = 11	Control group *n* = 11
	V/EVT index	V/EVT index
Histamine concentration (ng/1 g of tissue)	0,48	**0,74**
MCD (n/mm^2^)	0,03	**0,82**
MMCA (*μ*m^2^)	0,22	**0,67**
Shape index	0,51	0,42
